# Impact of Interleukin-6 on Oral Squamous Cell Carcinoma Among the South Indian Population

**DOI:** 10.7759/cureus.63789

**Published:** 2024-07-03

**Authors:** P Harini, Mahathi Neralla, Auxzilia Preethi, Sushmaa Chandralekha Selvakumar

**Affiliations:** 1 Oral and Maxillofacial Surgery, Saveetha Dental College and Hospitals, Saveetha Institute of Medical and Technical Sciences, Saveetha University, Chennai, IND; 2 RNA Biology Lab, Saveetha Dental College and Hospitals, Saveetha Institute of Medical and Technical Sciences, Saveetha University, Chennai, IND

**Keywords:** gene expression, tumor growth, oral squamous cell carcinoma, interleukin-6, biomarker

## Abstract

Introduction

Oral squamous cell carcinoma (OSCC) is associated with high rates of morbidity and mortality. Despite advances in research and treatment, the survival rate of OSCC patients has not changed considerably in recent years. Interleukin-6 (IL-6) is a proinflammatory cytokine that is involved in the development of various cancers including OSCC. The role of IL-6 is being studied in various cancers; however, its exact mechanism of action in OSCC among the South Indian population has not yet been studied. Thus, the current study aims to evaluate and assess the impact of IL-6 on OSCC among the South Indian population.

Materials and methods

Twenty tissues from OSCC patients and 20 normal tissues surrounding the same area from normal people were gathered from the Department of Oral and Maxillofacial Surgery, Saveetha Dental College and Hospital. The tissues were prepared for expression investigations and hematoxylin and eosin staining. The data was presented as mean ± standard deviation, with statistical significance at p<0.05.

Results

Our results indicate that, in comparison to normal tissues, OSCC samples had increased IL-6 expression levels (p<0.05).

Conclusion

We conclude that IL-6 has been identified as a key oncogene in the development of tumors and their spread in several types of cancers, including OSCC. Therefore, IL-6 can be used as a potential diagnostic or prognostic biomarker and the use of IL-6 inhibitors can be formulated as a potential treatment for OSCC.

## Introduction

Oral squamous cell carcinoma (OSCC) is one of the leading causes of cancer-related death globally, accounting for more than 95% of all head and neck cancers [1]. It is most common in men over the age of 45 who are exposed to various risk factors such as tobacco chewing, alcohol and pan consumption, and ultraviolet radiation. Identified causes include human papillomavirus (HPV), *Candida* infections, dietary inadequacies, and genetic predisposition [2]. In general, OSCC patients have a low five-year survival rate and a high recurrence rate. Several treatments, including surgery, chemotherapy, radiation, and immunotherapy, are used to treat OSCC, depending on the location and/or stage of the disease [3]. The multistep process of oral carcinogenesis is modulated by both endogenous and environmental factors. Tobacco chewing, regular alcohol intake, and the presence of HPV infection predispose a patient to the development of OSCC [4,5]. The constant presence of these predisposing factors leads to many genetic and epigenetic events in an individual, promoting genomic instability and tumor development and progression. The development and progression of premalignant and OSCC lesions are caused by irreversible DNA damage like gene deletion, mutation, and amplification, leading to activation or inactivation of oncogenes or tumor suppressor genes as well as epigenetic changes, which are changes in the gene expression not encoded in the DNA sequence [4,5]. Despite advances in research and treatment, the survival rate of OSCC patients has not changed significantly in recent years. Understanding the mechanisms underlying OSCC development and progression may lead to the discovery of novel diagnostic biomarkers and therapeutic compounds for OSCC treatment. Further discoveries regarding epigenetic changes hold the potential to revolutionize every aspect of cancer biology, which will offer preventative, diagnostic, and therapeutic advantages.

Cytokines play an important role in tumor microenvironment regulation and chronic pro-tumorigenic inflammation. They are soluble, low-molecular-weight polypeptides that are produced primarily by innate and adaptive immune system cells but also by resident tissue and tumor cells. They impact many aspects of cellular behavior, including growth, differentiation, and function. During inflammation and carcinogenesis, their physiological activities are disrupted [6]. The findings supported the role of proinflammatory cytokines in the development of cancers such as lung cancer, hepatocarcinoma, colorectal cancer, and OSCC. Interestingly, interleukin-6 (IL-6) can activate intercellular signal transmission and exchange by acting on cell receptors via autocrine or paracrine pathways, completing the necessary biological activities of cells [7]. The classical pathway and trans-pathway are the two transduction pathways for IL-6. According to studies, IL-6 is involved in tumor development, differentiation, apoptosis, immune response, and drug resistance [7,8]. The role of IL-6 is being studied in various cancers; however, its exact mechanism in OSCC of South Indian population has not yet been studied. Evaluating the expression levels of IL-6 could be helpful in understanding the development and progression of OSCC; therefore, the IL-6 expression analysis was carried out in the tissues of OSCC patients and compared with adjacent normal tissues in normal outpatients. Therefore, the objective of this research is to evaluate the impact of IL-6 on OSCC in the South Indian population.

## Materials and methods

Sample collection

Twenty patients diagnosed with OSCC and 20 adult outpatients without OSCC from the Department of Oral and Maxillofacial Surgery of Saveetha Dental College and Hospitals (SDCH), Chennai*, *India, were included in the study. Samples from both the subsets of patients were collected and processed. Informed consentwas signed by each participant prior to sample processing. For additional analysis, the materials were stored at -80°C.

Inclusion criteria

Patients who were diagnosed with histologically verified OSCC of any oral subsite were included in the study. The inclusion criteria also included patients being 18 years of age or older and capable of giving informed consent. The study also included 20 outpatients who did not have OSCC for comparison with normal tissues.

Exclusion criteria

Patients in whom oral submucosal fibrosis (OSMF), or any other premalignant lesion linked to OSCC, was determined by a clinical diagnosis were excluded. Individuals who declined to take part in the research were not considered. Individuals with OSCC of any other subsite in the head and neck were also not included.

 

Histopathological analysis

Before being fixed in paraffin, 10% formaldehyde was added to samples of cancerous tissues. The paraffin-embedded tissues were sectioned into 5 µm-thick slices and stained with hematoxylin and eosin (H & E). Then the tissue samples were examined under a light microscope [8].

RNA isolation and quantitative real-time PCR

Following the instructions from the manufacturer, total RNA from both the normal and malignant tissues was isolated using the TRIzol reagent (Invitrogen, Carlsbad, USA). The concentration and purity of the extracted RNA were assessed using a NanoDrop 2000 Lite spectrophotometer (Thermo Fisher Scientific, Waltham, USA). A collective 10 µl of total RNA was reverse transcribed into complementary DNA (cDNA) using Moloney murine leukemia virus (M-MLV) reverse transcriptase. The reaction mixture was incubated at 30°C for 10 minutes, 42°C for 30 minutes, 95°C for 5 minutes, and 4°C for the last incubation in a PCR (MiniAmp plus thermal cycler, Thermo Fisher, Waltham, USA) [9]. The extracted cDNA was quantified in Nanodrop Lite and kept cold until an additional examination was performed. After the cDNA was produced, expression experiments for the IL-6 gene were conducted using SYBR Green (Takara, Shiga, Japan), where the housekeeping control gene is GAPDH. The primer sequences for GAPDH (Fwd: 5'-ACCACCCTGTGGCTGTAGCCAA-3') and IL-6 (Fwd: AGACAGCCACTCACCTCTTCAG-3'; Rev: 5'-TTCTTGCCAGTGCCTCTTTGCTG-3') were determined. The samples were amplified and duplicated using the following thermocycling settings: denaturation at 95°C for 30 seconds, then 40 cycles of 5 seconds at 95°C and 30 seconds at 60°C. Finally, the 2-\^Cq technique was used to calculate the expression levels of IL-6 [10].

Statistical analysis

The information was displayed as mean ± standard deviation (SD). The student's t-test was used to compare the IL-6 levels in malignant and nearby normal tissues. A value of p < 0.05 was considered to be statistically significant.

## Results

Clinical characterization of participants

In our study, 16 individual samples were evaluated. The other samples were discarded due to contamination or insufficient sample size. The samples comprised 13 men and three women, with their age ranging from 30 to 60 years on average. Among them, 12 had a history of tobacco chewing and alcohol consumption habits. Four did not have any habits. Nine individuals had tumors in the T1 and T2 stages, while the remaining seven had tumors in the T3 and T4 stages. The samples consisted of eight well and eight moderately differentiated squamous cell carcinoma samples. Table 1 displays the clinical data pertaining to the samples.

**Table 1:** Clinical characteristics of the participants

Clinical Features		Total Cases (n = 16)
Age	< 50	11
	> 50	5
Gender	Male	13
	Female	3
Tobacco chewing habit	Yes	12
	No	4
Alcohol habit	Yes	12
	No	4
Histological grade	Well-differentiated	8
	Moderately differentiated	8
Tumor staging	T1-T2	9
	T3-T4	7
Tissue subsite	Right buccal mucosa	8
	Left buccal mucosa	8

Histopathological analysis of OSCC and normal tissue

**Figure 1:**
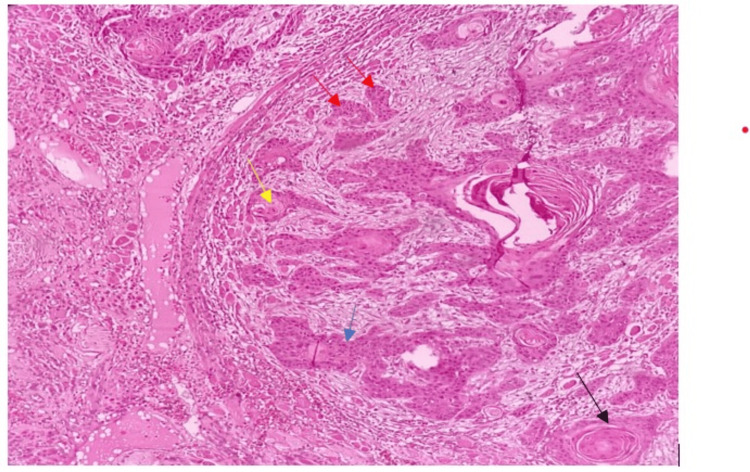
Slide demonstrating OSCC features. The black arrow shows the keratin pearl formation, the blue arrow shows the epithelial cells, the yellow arrow shows the nucleus, and the red arrow shows the islands of malignant epithelial cells.

In the OSCC areas, damaged epithelial cells were evident. During keratin synthesis, the nucleus and cytoplasmic organelles disappeared, causing tumor cell growth to accelerate and programmed cell death. Figure 1 displays the histological analysis of a well-differentiated squamous cell carcinoma.

IL-6 expression in normal tissues and OSCC tissues

**Figure 2:**
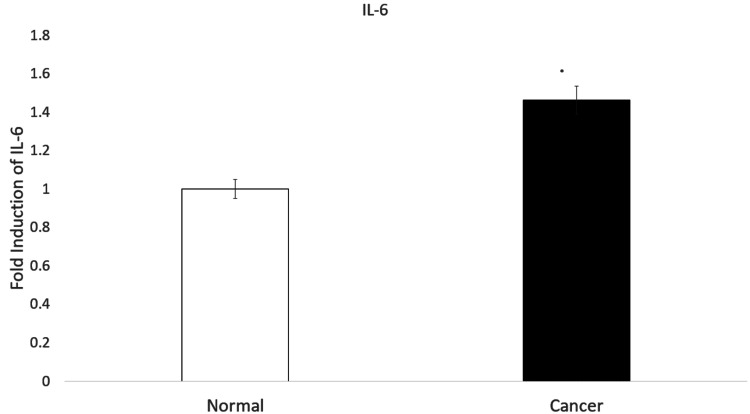
The expression levels of IL-6 in the OSCC and normal tissues. Data is shown as mean ± standard deviation;  p < 0.05 was considered statistically significant.

Figure 2 represents the expression level of IL-6.

Our results indicated that, compared to the surrounding normal tissue, the OSCC samples had considerably (p < 0.05) higher IL-6 expression levels.

## Discussion

In terms of systemic malignant tumors based on frequency, head and neck cancer ranks the sixth most common malignancy with reference to frequency and occurrence, with around 90% of the cases being OSCC with poor prognosis. However, it is unclear how OSCC develops [11]. On the one hand, persistent inflammation can draw inflammatory and immune cells to the tumor tissue, and as the tumor grows, these growth roles shift from immune monitoring and suppression to stimulating the growth of tumor cells. This is because chronic inflammation affects the NF-κB-IL-6-STAT pathway, which is crucial for tumor genesis, development, migration, and invasion. Interestingly, studies have shown that NF-κB is activated in OSCC and its downstream proinflammatory cytokines, such as interleukin-1 alpha , interleukin-8 , and tumor necrosis factor alpha, are overexpressed in tissues, serum, tissue-infiltrating lymphocytes, and cell lines of OSCC [12]. Antitumor adaptive immunity, which is the regulation of T cell priming and lymphoid trafficking in lymphoid organs, is achieved by activating IL-6 signalling [13]. IL-6 is also important for the differentiation of naive CD4+T cells into Th17 cells and to protect them from apoptosis [13]. 

A study by Mingyu Li et al. [14] suggested that IL-6 significantly induced ferroptosis resistance throughout the development of head and neck squamous cell carcinoma. The IL-6/STAT3/xCT axis functioned as a novel mechanism that promoted tumor development, and thus, IL-6 can be used as a target for tumor treatment and prevention [14]. Karakasheva et al. [15] reported that IL-6 promoted fibroblast activation to mediate crosstalk between tumor cells and cancer-associated fibroblasts. Consequently, the IL-6 receptor (IL6Rα) and downstream effectors present prospects for targeted therapy in upper gastrointestinal malignancies. In physiologically relevant 3D organotypic and tumoroid models as well as in mouse models of esophageal cancer, IL-6 depletion inhibited carcinogenesis. Thus, these findings supported the development of a potential treatment strategy to treat tumors by demonstrating the link between esophageal cancer and cancer-associated fibroblast by IL-6 signaling [15]. Márton et al. [16] suggested that age and dental health were substantially correlated with the expression of salivary IL-6 mRNA. IL-6 expression was found in tumor cells and tumor-infiltrating leukocytes, indicating the presence of a paracrine stimulation loop. Thus, salivary IL-6 mRNA is among the most effective and clinically important OSCC biomarker [16]. IL-6 promotes an immunosuppressive tumor environment by contributing to the expansion of derived suppressor cells and regulatory T cells, thereby inhibiting the antitumor immune response [17].

IL-6 is also upregulated in a variety of other cancers like pancreatic cancer, non-small-cell lung cancer, breast cancer, ovarian cancers and melanoma. IL-6 is also associated with events necessary for the development of secondary tumors or metastasis [18]. It is reported to be two-fold to ten-fold higher in cancer tissues than in noncancer tissues [18]. Upregulation also was seen to be correlating with advanced disease stage, poor prognosis, and decreased overall survival [18,19]. In the present study, it was found that the OSCC samples had considerably (p < 0.05) higher IL-6 expression levels than the adjacent normal tissue.

The main limitation of our study is the sample size. More studies done with larger sample sizes will make the results more conclusive. The underlying IL-6-mediated tumorigenesis in OSCC needs more research. The main advantage of our study is that it is one of the few studies focusing on the South Indian gene pool. The heterogenicity of OSCC and genetic variations in different gene pools make studies focused on single gene pools essential for precision diagnostics and cancer care.

## Conclusions

IL-6 had an impact on OSCC cells; however, the exact mechanism of the onset and progression of OSCC has yet to be studied in various signaling pathways. The levels of IL-6 can be reduced using IL-6 inhibitors, namely anti-IL-6 receptor monoclonal antibodies (e.g., tocilizumab, sarilumab, and siltuximab). To propose these proinflammatory IL-6 cytokines as a screening or diagnostic markers for regular use in clinical practice, we need studies with larger sample sizes, animal models, and OSCC-related cell line studies.
